# The association between dual sensory impairment and dementia: a meta-analysis and systematic review of the literature

**DOI:** 10.1093/ageing/afaf267

**Published:** 2025-09-27

**Authors:** Nina Meret Zumbrunn, Karen Beckett, Johannes Alfons Karl, Fiona N Newell, Louise Hopper, David P McGovern

**Affiliations:** School of Psychology, Dublin City University, Glasnevin Campus Dublin, Dublin 9, Ireland; School of Psychology, Dublin City University, Glasnevin Campus Dublin, Dublin 9, Ireland; Stanford University Graduate School of Business, Stanford University, 655 Knight Way, Stanford, CA 94305-7298, California, USA; School of Psychology and Institute of Neuroscience, Trinity College Dublin, Dublin 2, Ireland; School of Psychology, Dublin City University, Glasnevin Campus Dublin, Dublin 9, Ireland; School of Psychology, Dublin City University, Glasnevin Campus Dublin, Dublin 9, Ireland

**Keywords:** dual sensory impairment, dementia, sensory impairment, vision impairment, hearing impairment, systematic review, older people

## Abstract

**Background:**

Recent evidence suggests an association between dual sensory impairment (DSI)—that is, both visual and hearing impairments—and dementia. The aim of this systematic review was to synthesise the literature evaluating the dementia risk of adults >18 years with DSI compared to those without sensory impairment and/or those with a single sensory impairment (SSI).

**Methods:**

PubMed, Web of Science and PsycINFO were systematically searched in February 2024 and August 2024 for studies that considered the association between DSI and dementia, and compared individuals with DSI to those with either no sensory impairment or a SSI. A meta-analysis was conducted on studies reporting hazard ratios. The review followed Preferred Reporting Items for Systematic Reviews and Meta-Analysis guidelines and was registered on the Prospective Register of Systematic Reviews (PROSPERO).

**Results:**

A total of 22 papers were included in the narrative review, and 13 were included in the meta-analysis. Overall, findings from the narrative synthesis highlighted a consistent link between dementia prevalence and incidence with DSI. The meta-analysis revealed that individuals with DSI are at an ~50% greater risk of developing dementia compared to those without sensory impairments. Moreover, we identified DSI as a potentially greater risk factor for dementia than isolated hearing and visual impairment, with the risk for dementia in the DSI group exceeding the combined risk of the individual sensory impairments.

**Conclusions:**

This review highlights that there is substantial evidence linking DSI to an increased risk of dementia, emphasising the importance of considering the interplay between multiple senses in dementia research. Future research should focus on exploring whether interventions targeting DSI could also improve cognitive outcomes.

## Key Points

- Dual sensory impairment (DSI) is a modifiable risk factor for dementia.
- DSI increases dementia risk by ~50% compared to those without sensory impairments.
- The dementia risk of DSI exceeds the additive dementia risk of single impairments

## Introduction

Dementia affects >50 million people worldwide, with the prevalence projected to reach >150 million in 2050 [1]. Due to this rapid growth, researchers have increasingly focused on identifying early markers indicating an increased dementia risk before thresholds for a clinical diagnosis are reached [2]. A recent estimate suggested that ~384 million people >50 years are currently in the preclinical or mild cognitive impairment (MCI) stage of Alzheimer’s disease, the most common type of dementia [3]. This highlights the need to identify modifiable markers that indicate the presence of dementia or pre-dementia stages as early as possible to enable early intervention [4]. Moreover, modifiable risk factors may themselves be targets for intervention and may optimise the benefits of pharmacological treatments that delay cognitive decline [5, 6].

In aiming to identify potential risk factors for dementia, researchers have considered the role of sensory impairments, mainly focusing on visual impairment (VI) or hearing impairment (HI) separately. Indeed, multiple systematic reviews and meta-analyses have synthesised evidence for an association between HI or VI and dementia risk [7–11]. Moreover, the most recent *Lancet Commission* report, now includes both VI and HI in a list of modifiable risk factors that could prevent up to 45% of dementia cases [12]. The association between HI or VI and dementia is especially alarming considering that the World Health Organization reports at least 2.2 billion people currently living with VI [13] and nearly 2.5 billion of the global population have HI [14]. Moreover, the prevalence of HI and VI not only increases with age, but is also predicted to grow rapidly within the next decades [15, 16].

The link between sensory impairment and cognitive decline becomes more complex when considering that multiple sensory impairments may occur simultaneously. Of particular significance is dual sensory impairment (DSI) consisting of concurrent HI and VI. Studies have reported that up to 21% of individuals may experience DSI [17, 18]. Furthermore, DSI prevalence increases with age [19], and is also associated with other common dementia risk factors such as depression [20]. It is therefore unsurprising that there is a growing consensus that DSI is a risk factor for dementia [21–24] and that DSI may even pose a greater risk than single sensory impairment (SSI) consisting of VI or HI only [25, 26]. Thus, the aim of this review is to consolidate and synthesise evidence from studies examining the relationship between DSI and dementia to provide a comprehensive understanding of the potential of DSI to act as a marker of dementia. Furthermore, we will conduct a meta-analysis to compare the association between DSI and dementia to that of SSI and dementia, to understand whether DSI might be a greater risk factor. In doing so, we will also explore the impact of different measurements of sensory impairments on this association; that is, potential differences between subjective and objective assessment of sensory impairment.

## Methodology

This review follows the Preferred Reporting Items for Systematic Reviews and Meta-Analysis framework (PRISMA 2020) [27]. The review is registered with PROSPERO (ID: CRD42024507724). The PICOS tool was used to determine inclusion and exclusion criteria. We included studies on adults with DSI, consisting of HI and VI, whose objective dementia status was compared to a group of participants with NSI or SSI. Assessment of sensory function could be subjective or objective and sensory aids were included. Any study incorporating cognitive interventions was excluded. Included study designs consisted of peer-reviewed articles published in English. Books, reviews, letters, case studies, conference abstracts, protocols, editorials and animal studies were excluded.

Database searches were conducted on the 29 February and 30 August 2024 on PsycINFO, PubMed and Web of Science. All studies were uploaded to Covidence where duplicates were removed and both abstract and full-text screening were conducted by two authors independently (NMZ, KB). Data extraction and quality appraisal [28] was performed by NMZ.

Studies including hazard ratios (HRs) and 95% confidence intervals (CI) were included in a meta-analysis. For studies reporting multiple models with covariates included, we used the HR derived from the model with the most covariates included. Standard errors were estimated from the 95% CIs, and the log transformation of HRs was conducted for normalisation of the distribution. To test for heterogeneity, Cochrane’s Q test, as well as the I^2^ statistic were calculated, with results <50% indicating moderate heterogeneity [29]. Furthermore, to compare the dementia risk between DSI, HI and VI groups, the relative excess risk due to interaction (RERI) was calculated using the following equation [30]:


$$ \mathrm{RERI}=\mathrm{HRDSI}-\mathrm{HRHI}-\mathrm{HRVI}+1 $$

Sensitivity analyses were conducted to examine the influence of each study on the overall findings by sequentially excluding individual studies from the model. Publication bias was assessed by examining funnel plots. Forest plots were created to illustrate the individual and pooled effect size of all studies included in the meta-analysis. Studies that did not report HRs were excluded from the meta-analysis. Instead, these studies were summarised through narrative synthesis.

A more detailed description of the methodology can be found in Supplementary Materials (Appendix 1).

## Results

### Literature search

A total of 1040 references were retrieved across databases. After removal of duplicates, 670 abstracts were screened. In total, 136 articles were deemed to be relevant and proceeded to full text review. Overall, 22 studies were included in the systematic review (see Supplementary Materials, Appendix 2) [21, 23–26, 31–47].

### Overall study characteristics

The included studies were published between 1991 and 2024, with most published between 2021 and 2024 (*n* = 13). Most studies were conducted in North America (*n =* 9), followed by Asia (*n =* 5) and Europe (*n =* 5). Three studies were based in multiple European countries. Overall, 16 studies used a longitudinal design, whereas six studies were cross-sectional. The sample size ranged from 174 to 352 656 participants, whilst mean age ranged from 56.8 to 83.4 years.

### Prevalence of sensory impairment in objective versus subjective measurements

**Figure 1:**
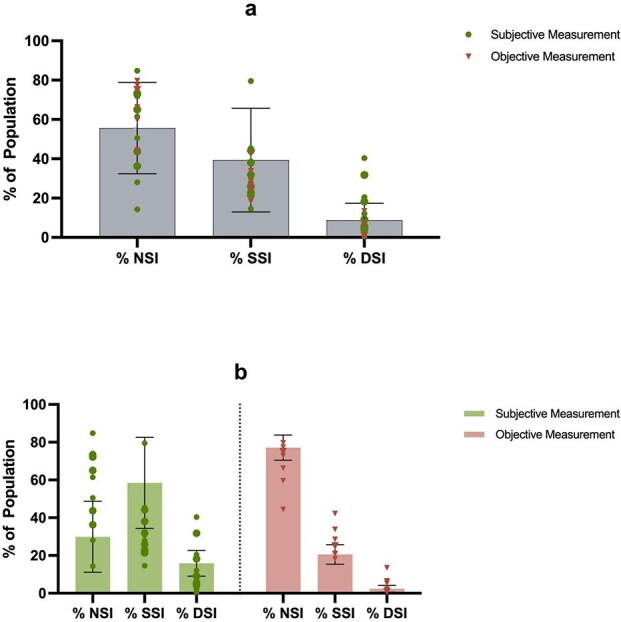
Overview of prevalence of no sensory impairment (NSI), SSI and DSI. (a) Illustrates the overall weighted mean percentage of sensory impairment prevalence across studies, with individual points representing studies, which were either subjective (circle shape) or objective (triangle shape). (b) Illustrates weighted mean percentage of sensory impairment prevalence of studies measuring sensory impairment subjectively (green bar with dots indicating individual studies) or objectively (red bar, with triangles indicating individual studies), with individual points representing prevalence of individual studies. Error bars above and below the mean indicate standard error of the mean.

Of the 1 160 824 participants included across the studies, 102 120 had DSI (weighted average = 8.79%, SD = 8.57). DSI prevalence was higher among studies that used subjective (weighted average = 15.90%, SD = 6.83) compared to objective sensory measurements (weighted average = 2.40%, SD = 1.75). Similarly, the mean average of SSI (overall weighted average = 39.34%, weighted SD = 26.35) was higher when SSI was measured subjectively (weighted average = 58.46%, SD = 24.08) compared to objective measurements (weighted average = 20.54, SD = 5.20). Across all studies 55.62% of participants did not have a sensory impairment (SD = 23.23) (see Figure 1). Additional analysis of prevalence of sensory impairments in participants with dementia as well as a synthesis of sensory measurement types can be found in Supplementary Materials (Appendices 3 and 4).

### Dementia

#### Study characteristics and assessments

Dementia was most commonly assessed using diagnostic criteria, including the International Classification of Diseases (ICD) (*n* = 5), or the Diagnostic and Statistical Manual of Mental Disorders (DSM) (*n* = 3). Three studies used self-reported physician diagnosis. The remaining studies used cognitive assessments and interviews or neuropsychological tests to determine the presence of dementia (see Table 1 for overview).

**Table 1:** Overview of study characteristics.

Author (date)	Country	Study design	*n* (end point)	Mean age (SD)	Outcome measurement	Covariates	VI measurement	VI cut off	HI measurement	HI cut off	DSI definition
Assi *et al.* (2021)	United States	CS	7124	Not reported, participants were >65 years old	Physician diagnosis, AD8 dementia screening interview score ≥ 2, score ≤ 1.5SD on at least 1 cognitive test	N/A	Self-report (accuracy checked by assessor)	Blindness, not being able to see across the street and/or read newspaper print despite corrective aid use	Self-report (accuracy checked by assessor)	Deafness, hearing aid use, not being able to hear well enough to use the telephone or to carry on a conversation in a room with a radio or TV playing	Presence of HI and VI according to criteria
Byeon *et al.* (2021)	South Korea	Both	6520 (3818)	69.6 (not reported)	DSM-IV	Age, sex, education, income, modified cumulative illness rating scale, BMI, alcohol, smoking, physical activity, depression, social support	Self-report	Problems reading newspaper or watching TV regardless of corrective aid use, blindness	Self-report	Difficulty communicating regardless of hearing aid use, deafness	Presence of HI and VI according to criteria
Davidson & Guthrie (2019)	Canada (Ontario)	CS	352 656	82.8 (7.9)	CPS, not further specified	None	Self-report (accuracy checked by assessor)	Not reported	Self-report (accuracy checked by assessor)	Not reported	Score of ≥3 on deafblind severity index
Deardorff *et al.* (2019)	United States	CS	24 009	79.15 (not reported)	ICD-9	Age, sex, race, income, living status, Medicaid eligibility, chronic conditions	Self-report	Self-reporting ‘a lot of trouble’ seeing	Self-report	Self-report ‘a lot of trouble’ hearing	Presence of HI and VI according to criteria
Dintica *et al.* (2023)	Sweden (Stockholm)	L	2579 (1260*)	Not reported, participants were >60 years old	DSM-IV-TR	Age, sex, education, smoking, alcohol, diabetes, cardiovascular disease, cerebrovascular disease, ADL limitations, depression	Jaeger Eye chart	≥ J2 averaged across both eyes	Medical files with ICD code, self-report	Self-report trouble hearing, use of hearing aid, presence of ICD codes	Presence of HI and VI according to criteria
Hu *et al.* (2022)	United Kingdom	L	113 511	56.8 (8.09)	ICD-9, ICD-10	Age, gender, ethnicity, Townsend index, obtainable education, physical activity, depression, overall health status	logMAR chart	Less than 0.3 logMAR in better seeing eye	Digit Triplet Test	Less than −5.5 dB speech reception threshold in better ear	Presence of HI and VI according to criteria
Hwang *et al.* (2020)	United States	L	3069 (2051)	78.66 (not reported)	ICD-IV	Age, sex, race, education, income, BMI, alcohol, smoking, physical activity, cardiovascular disease, cerebrovascular disease, diabetes, hypertension, clinic site, treatment status, APOE	Self-report	Inability to drive, watch TV, read newspaper or recognise someone across the room, regardless of corrective aid use	Self-report	Inability to hear the radio, use the telephone or hold a conversation in a crowded room, regardless of hearing aid use	Presence of HI and VI according to criteria
Hwang *et al.* (2022)	United States	L	5888 (2927)	74.5 (4.8)	DSM-IV	Age, sex, race, education, income, BMI, alcohol, smoking, physical activity, cardiovascular disease, cerebrovascular disease, diabetes, hypertension, clinic site, treatment status, APOE	Self-report	Inability to drive, watch TV, read newspaper or recognise someone across the room, regardless of corrective aid use	Self-report	Inability to hear the radio, use the telephone or hold a conversation in a crowded room, regardless of hearing aid use	Presence of HI and VI according to criteria
Kim *et al.* (2024)	Japan	L	14 186 (14,186)	80.4 (7.5)	ICD-10	Age, sex, long-term care needs, self-reliance levels, diabetes, cancer, ischemic heart disease, cerebrovascular disease, heart failure, arthritis, fracture	Medical diagnosis	ICD-10 vision disorders	Medical diagnosis	ICD-10 hearing disorders	Presence of HI and VI according to criteria
Kuo *et al.* (2021)	United States	Both	8245 (7562)	Not reported, participants were >65 years old	IWR, DWR, clock drawing test, name the date, month, year and the president/vice president of the US	Age, sex, education, race/ethnicity, smoking, hypertension, stroke, heart attack, heart disease, lung disease, cancer	Self-report	Inability to see well enough to read newspaper, recognise someone across the street, or blindness in regular aided conditions	Self-report	Hearing aid use, deafness, inability to hear well enough to use the telephone or carry on a conversation in a room with the TV or radio playing	Presence of HI and VI according to criteria
Li *et al.* (2024)	United States	L	20 248 (20,248)	66.7 (11.0)	Self-reported physician diagnosis	Age, sex, race, education, marital status, smoking, alcohol, physical activity, hypertension, diabetes, heart disease, stroke	Self-report	Fair or poor eyesight even when using glasses	Self-report	Fair or poor eyesight even when using hearing aids	Presence of HI and VI according to criteria
Luo *et al.* (2018)	China	CS	250 752	Not reported, participants were >65 years old	ICD-10	Age, sex, marital status, residence, region, education level, annual family per capita income	Not reported	Best-corrected visual acuity ≤0.05, visual field <10 degrees in the better seeing eye	Pure tone audiometry	More than 40 dB hearing loss in the better ear	Presence of HI and VI according to criteria
Maharani *et al.* (2020)	United States	L	19 681	57.8 (6.2)	Telephone Interview for Cognitive Status	Age, gender, marital status, education, income, smoking, alcohol, physical activity, chronic diseases, depression	Self-report	Fair or poor vision in regular aided conditions	Self-report	Fair or poor vision in regular aided conditions	Presence of HI and VI according to criteria
Maruta *et al.* (2020)	Japan	L	2190	78.9 (6.1)	Dementia Scale	Sex, care-need level, degree of independent daily living for disabled older adults.	Assessor report	Only being able to see vision chart at a distance of 1 m or less, or having very poor eyesight in regular aided condition	Assessor report	Being able to only hardly hear normal conversation, loud conversation or can hardly hear at all	Presence of HI and VI according to criteria
Michalowsky *et al.* (2019)	Germany	L	122 708	80.7 (6.8)	ICD-10	Age, sex, health insurance coverage, diabetes mellitus, ischemic heart disease, stroke, intracranial injury, epilepsy, Parkinson’s disease, depression	Medical files	ICD-10 diagnosis of H25-H28, H30-H36, H43-H45, H46-H48, H49-H52, H53, H54	Medical files	ICD-10 diagnosis of H90 or H91	Presence of HI and VI according to criteria
Möller *et al.* (2024)	Denmark, Sweden, Austria, Germany, the Netherlands, France, Switzerland, Belgium and Luxembourg, Spain, Italy, Greece and Portugal. Czech Republic, Poland, Hungary, Slovenia, Estonia and Croatia.	L	72 287 (72,287)	63.9 (9.6)	Self-reported physician diagnosis	Age, wave, region, partner in household, education, household income, medical history	Self-report	Fair or poor ability to read ordinary newspaper print, or recognising a friend across the street	Self-report	Poor or fair hearing	Presence of HI and VI according to criteria
Oh *et al.* (2023)	South Korea (Seoul)	L	202 547 (55,800)	64.8 (not reported)	ICD-10	Comorbidity score, alcohol, smoking, physical activity	Snellen chart	Less than 0.3 logMAR in both eyes whilst wearing correction	Not reported	More than 40 dB in both ears without hearing aid	Presence of HI and VI according to criteria
Pabst *et al.* (2021)	Germany	L	3497	79.8 (3.9)	SIDAM (incl. DSM-IV and DSM-III-R)	Age, gender, marital status, school education, alcohol consumption, smoking status, cognitive functioning, cardiac diseases, stroke/TIA, depression, diabetes mellitus	Self-report	Slight, moderate, or severe/profound impairment in seeing	Self-report	Slight, moderate, or severe/profound impairment in hearing	Presence of HI and VI according to criteria
Shi *et al.* (2024)	United Kingdom	L	68 305 (68,305)	Not reported, participants were between 39 and 70 years old	Algorithmically defined outcomes	Age, gender, income level, education, smoking, alcohol, socioeconomic status, hypertension, cardiovascular disease, diabetes, physical activity, APOE4 status	logMAR chart	Less than 0.3 logMAR	Self-report	Experience of any HI or deafness	Presence of HI and VI according to criteria
Uhlmann *et al.* (1991)	United States (Seattle)	CS	174	77 (0.5)	National Institute of Neurologic and Communicative Disorders and Stroke/Alzheimer’s Disease and Related Disorders Association criteria for the clinical diagnosis of ‘probable’ Alzheimer; MMSE	Family history of dementia, depression, number of prescription drugs	Snellen chart, Rosenbaum chart	Visual acuity less than median in control group	Pure tone audiometry	More than 30 dB pure-tone average	Presence of HI and VI according to criteria
Yamada *et al.* (2014)	Czech Republic, England, Finland, France, Germany, Israel, the Netherlands	CS	4007	83.4 (9.4)	Not reported	N/A	Self-report	Inability to see newspaper headlines, to identify objects or blindness	Self-report	Difficulty hearing if not in quiet setting, or in all setting or deafness	Presence of HI and VI according to criteria
Yamada *et al.* (2016)	Czech Republic, England, Finland, France, Germany, Israel, the Netherlands	L	1989 (1443)	83.3	Not reported	N/A	Self-report	Inability to see newspaper headlines, to identify objects or blindness	Self-report	Difficulty hearing if not in quiet setting, or in all setting or deafness	Presence of HI and VI according to criteria

^*^L = longitudinal, CS = cross-sectional.

### The association between dual sensory impairment and dementia

Overall, 18 of 22 studies found a higher likelihood of dementia in participants with DSI [21, 24–26, 32–36, 38–47]. Of nine studies that examined dementia prevalence cross-sectionally, six found a higher likelihood of dementia in those with DSI [24, 33, 35, 36, 44, 45]. One study reported significant group differences in dementia prevalence, with the highest prevalence in the DSI group, although pairwise comparisons were not reported [32]. Two studies found no association between DSI and dementia prevalence [23, 31]. Interestingly, one study found an association between DSI and dementia prevalence after adjusting for demographic and age factors, but this association was no longer significant when depression and social support were included [31].

Of the nine studies evaluating the association between DSI and dementia prevalence, six also examined SSI. Two studies found an association between SSI and dementia; however, the association between DSI and dementia was reported to be stronger in both studies [35, 44]. Four studies did not find an association between SSI and dementia prevalence [24, 31, 33, 36] (see Table 2 for overview).

**Table 2:** Overview of study findings of association between dementia prevalence and DSI according to type of sensory function and dementia measurement.

Outcome	Author	Subjective HI	Subjective VI	Diagnostic dementia	Increased risk for DSI	Increased risk for SSI	Greater Risk in DSI compared to SSI
Dementia prevalence	Byeon *et al.* 2021	**✓**	**✓**	**✓**			
	Davidson & Guthrie, 2019	**✓**	**✓**		**✓** ^a^		
	Deardorff *et al.* 2019	**✓**	**✓**	**✓**	**✓**		
	Kuo *et al.* 2021	**✓**	**✓**		**✓**	**✓**	**✓**
	Luo *et al.* 2018			**✓**	**✓**		
	Uhlmann *et al.* 1991			**✓**	**✓**	**✓**	**✓**
	Yamada *et al.* 2014	**✓**	**✓**		**✓**	0	
	Yamada *et al.* 2016	**✓**	**✓**			0	
Dementia incidence	Byeon *et al.* 2021	**✓**	**✓**	**✓**			
	Dintica *et al.* 2023			**✓**	**✓**		
	Hu *et al.* 2022			**✓**	**✓**	**✓**	**✓**
	Hwang *et al.* 2020	**✓**	**✓**	**✓**	**✓**		
	Hwang *et al.* 2022	**✓**	**✓**	**✓**	**✓**	**✓**	**✓**
	Kim *et al.* 2024				**✓**		
	Kuo *et al.* 2021	**✓**	**✓**		**✓**	**✓**	**✓**
	Li *et al.* 2024	**✓**	**✓**	**✓**	**✓**		
	Maharani *et al.* 2020	**✓**	**✓**			**✓**	
	Maruta *et al.* 2020	**✓**	**✓**	**✓**	**✓**	**✓** ^b^	**✓**
	Michalowsky *et al.* 2019			**✓**	**✓**	**✓** ^b^	**✓**
	Möller *et al.* 2024	**✓**	**✓**	**✓**	**✓**	**✓**	**✓**
	Oh *et al.* 2023			**✓**	**✓**	**✓**	**✓**
	Shi *et al.* 2024				**✓**	**✓**	(**✓**)^c^

^a^Greater risk for dementia other than AD in comparison to those without DSI (SSI+NSI)

^b^Only HI, but not VI was associated with greater risk for dementia.

^c^DSI was only associated with a greater risk for all-cause dementia compared to HI; however, the risk for all-cause dementia was greater in those with VI only.

0 = not analysed.

Of the 22 included studies, 14 considered the association between DSI and dementia incidence. Of those, 12 found that DSI increases likelihood of dementia longitudinally [21, 25, 26, 34, 35, 38–41, 46, 47], whilst two studies found no association [31, 37].

Thirteen of the 22 studies also evaluated the relationship between SSI and dementia. Nine studies found a higher likelihood of dementia in individuals with SSI [26, 34, 35, 37–41, 46] and two studies found a higher likelihood of dementia in HI but not VI [38, 39]. Of these 11 studies, eight reported DSI to be a greater risk factor for dementia than SSI [26, 35, 38–41, 43, 46]. One study found an association between SSI and incident dementia but not DSI [37] (see Table 2). Further analyses on the association between DSI and specific dementia subtypes as well as sensory impairment and dementia prevalence can be found in the Supplementary Materials (Appendices 4 and 5).

#### Variables influencing the relationship

Two studies found that social factors, such as social support and leisure activity, influenced the DSI-dementia relationship [25, 31]. Four studies found that the association between DSI and dementia was only significant in APOE e4 non-carriers [21, 25, 31, 46]. Four studies found that DSI was associated with dementia across age groups [25, 34, 40, 47]. However, whilst two studies reported that the risk of all-cause dementia seemed to increase with age for individuals with DSI [21, 25] one study reported a higher risk in the younger age group [46]. Additionally, it was reported that DSI was only significantly associated with dementia in female participants [21]. Interestingly, one study reported that dementia risk increased in the 50–64 and 65–79 age groups for male participants with DSI, whilst this decreased in the same age groups for females with DSI. However, the risk for dementia was greater in females with DSI aged 50–64 years compared to males with DSI in that age group [40]. However, another study reported no sex differences in the relationship between DSI and dementia [34]. One study reported an association only in those with severe dementia [36]. Another study reported that risk for dementia increases with DSI severity [21]. Notably, one study reported that the association between DSI and dementia was only significant for participants who had DSI for >2 years. More specifically, they noted an increase of all-cause dementia risk by 31% and a 46% increase for ad risk per year with DSI [46].

### Meta-analysis

**Figure 2:**
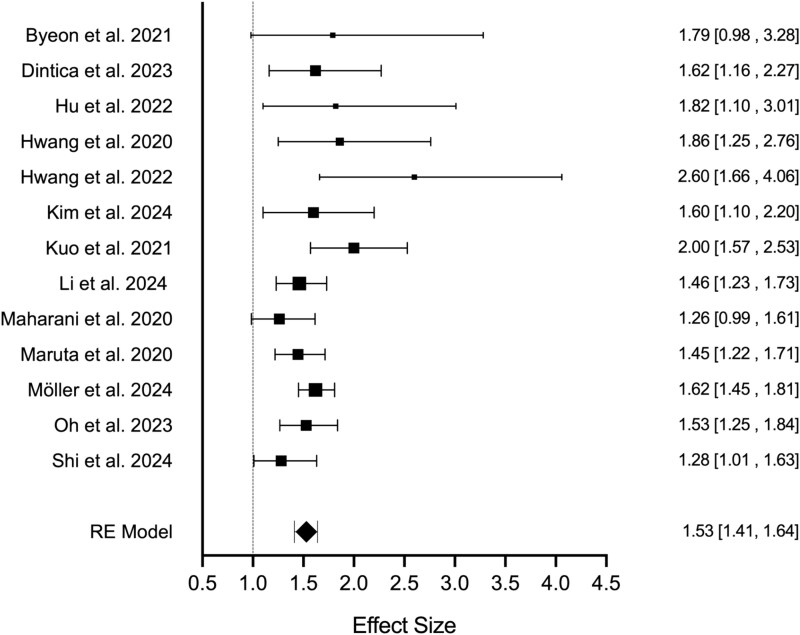
Forest plot illustrating the individual study and overall effect sizes of dementia hazard in participants with DSI. The dotted line represents no change in the hazard for dementia, whilst values greater than one indicate an increased risk.

A meta-analysis was conducted on 13 studies that reported HRs and CIs, [21, 25, 26, 31, 34, 35, 37, 38, 40, 41, 43, 46, 47]. The analysis included 383,326 participants. Heterogeneity was low to moderate across studies (I^2^ = 32.75%), with no evidence of publication bias based on funnel plot inspection and Egger’s test (*P* > .05). The random effects analysis revealed that individuals with DSI had a moderately greater risk of dementia compared to those with NSI (HR = 1.53, 95% CI 1.41 to 1.64, see Figure 2). Subgroup analysis focusing on subjective sensory assessment revealed an increased HR (HR = 1.62, 95% CI 1.36 to 1.88), though increased heterogeneity warrants caution in interpreting this finding (I^2^ = 54.58%). Limiting the analysis to only include objective assessments of sensory function, revealed a slight reduction in the HR (HR = 1.52, 95% CI 1.41 to 1.64), with a substantial reduction in heterogeneity (I^2^ = 0%). Furthermore, excluding studies without clinically verified diagnosis (e.g. DSM, ICD) increased the hazard for dementia in those with DSI (HR = 1.63, 95% CI 1.49 to 1.77) and reduced heterogeneity (I^2^ = 0%). Lastly, limiting analysis to studies with follow-up times >5 years also slightly raised the hazard (HR = 1.56, 95% CI 1.40 to 1.71). Sensitivity analyses confirmed that no single study or group of related studies significantly affected these results.

**Figure 3:**
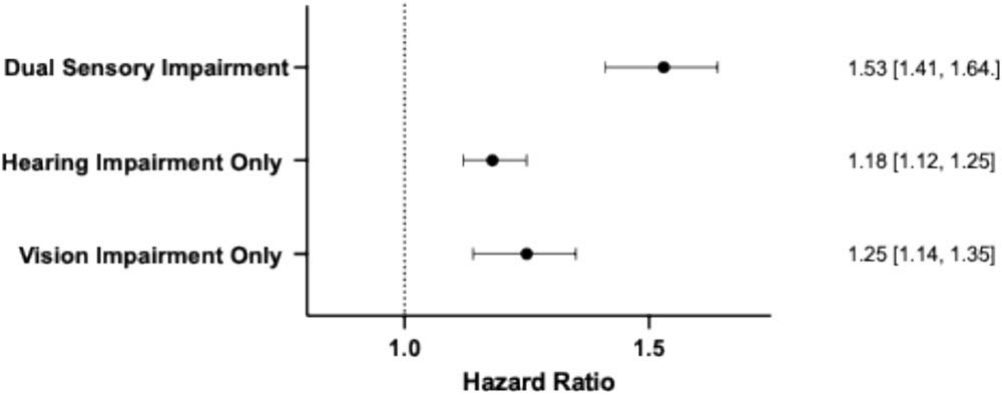
Forest plot illustrating dementia HR in participants with DSI, with HI only and vision impairment only. All groups are compared to participants with NSI as a reference group. The dotted line represents no change in the hazard for dementia, whilst values greater than one indicate an increased risk.

To determine whether DSI poses a potentially greater dementia risk than SSI, we conducted t-tests on the HRs from studies that included DSI, VI and HI groups. We found that HI was associated with a significant increase in dementia risk (HR = 1.18, 95% CI 1.12 to 1.25) compared to individuals with NSI. However, this risk was significantly smaller than the dementia risk of participants with DSI [t(22) = 5.45, *P* < .001, two-tailed]. Similarly, VI alone was associated with dementia risk (HR = 1.25, 95% CI 1.14 to 1.35) compared to NSI (see Figure 3). However, the heterogeneity for this analysis was substantial (I^2^ = 72.09%), warranting caution in interpretation. Nevertheless, the HR for DSI was significantly greater than for VI when compared to NSI [t(22) = 3.582, *P* < .001, two-tailed].

Lastly, we assessed additive interactions between HI and VI using the RERI, providing insight into whether the combined effect of the two SSIs on dementia risk exceeds the sum of their individual effects. We found a RERI of 0.01, indicating a slight supra-additive interaction—suggesting that DSI may pose a greater dementia risk than the sum of HI and VI alone. However, due to the small magnitude of the RERI this finding should be interpreted with caution.

### Quality assessment

Using the quality appraisal tool [28], all articles were rated to be of good quality. However, a lack of statistical justification of sample size of studies with smaller sample sizes, as well as a lack of validation of self-report measurement of sensory function was evident. Moreover, some studies failed to report whether participants wore corrective aids during sensory assessments. Lastly, whilst most studies identified limitations, only a few considered the impact of sensory impairment on the performance of cognitive tests that use auditory instructions, or visual tasks. Furthermore, there was a lack of consideration of comparing participants with and without corrective aids, as well as the consideration of sensory impairment onset.

## Discussion

This review aimed to evaluate whether DSI is associated with a higher risk of dementia compared to NSI or SSI. Synthesising data from 22 studies revealed a common association between DSI and both dementia prevalence and incidence. In line with these findings, a meta-analysis of HRs from 13 of these studies indicated an increased hazard of dementia in the DSI group relative to individuals with NSI. The strong association between DSI and dementia revealed in this review indicates that future research should consider sensory function more holistically, rather than focusing on impairments in isolation. This is further supported by our finding of significantly higher dementia hazard associated with DSI than the dementia hazard of VI and HI alone when these populations were compared to groups with NSI. This indicates that DSI may pose a greater dementia risk than SSI. Additionally, we found that the dementia risk for individuals with DSI is greater than the sum of the individual effects of HI and VI; however, this finding should be interpreted with caution due to its small magnitude.

Our findings reinforce the importance of prioritising a holistic approach to sensory impairment in dementia research, particularly in the context of developing effective interventions. More specifically, as DSI is a ‘modifiable’ risk factor for dementia, future research should explore whether the use of sensory aids might mitigate dementia risk for the DSI population. In support of this view, previous research has highlighted that hearing aids [48, 49], vision aids [22] and cataract surgery [50] can reduce dementia risk and cognitive decline. However, none of the studies included in this review grouped participants based on sensory aids use or conducted any adjustments based on sensory aids use of participants. Thus, future research should explore the impact of sensory aids on the link between DSI and dementia.

Investigating sensory aids as interventions for dementia among individuals with DSI may also help clarify the mechanisms linking DSI and dementia. Specifically, evidence of reducing dementia risk through the use of sensory aids would suggest a causal link between the two factors. For instance, it has been suggested that sensory loss could directly increase dementia risk, through an impoverished sensory environment causing a decrease in cognitive stimulation [51]. This in turn might negatively impact brain structure and function, thereby increasing the risk of dementia [51]. For example, HI has been linked to brain volume loss which could be explained by decreased sensory stimulation [52, 53]. According to this theory, an increased dementia risk in DSI may be explained by an aggregated loss of brain function and structure. However, an alternative hypothesis is that sensory loss and dementia may be mediated by other factors, such as social isolation—which is increased by sensory loss and a known risk factor for dementia [54–56]. Two studies in our review provide support for this theory showing that social factors influence the link between DSI and dementia [25, 31]. Similarly, DSI prevalence increases with age [19], and age itself is considered a risk factor for dementia [57].

Whilst the presence of DSI prior to dementia diagnosis suggests possible causal or mediated pathways, preclinical dementia neuropathology may already be present at baseline even if dementia was not yet diagnosable [58]. This is especially important, given the variability of follow-up times of the included studies. As such, it has been proposed that sensory loss and dementia are not directly linked, but share common mechanisms, such as underlying neuropathological processes [51]. For instance, common mechanisms may affect brain structure related to the development of dementia whilst also affecting structures relevant for hearing or vision such as the retina or cochlea [59, 60]. In this case, DSI may be considered a symptom of underlying neurodegeneration and sensory intervention may not reduce dementia risk. Future research should use longitudinal designs to explore these mechanisms, incorporating variables such as social isolation, sensory aid use, as well as potential use of brain imaging to observe neuropathological processes.

When investigating the longitudinal association between DSI and dementia, a critical, but largely overlooked factor is the dynamic nature of sensory function. That is, throughout the course of a longitudinal study, sensory loss may newly develop, worsen or improve with the use of interventions. Indeed, one longitudinal study assessing the relationship between general cognition and sensory function reported that at follow-up almost 35% of participants reported a new sensory impairment [61], although it was not considered whether sensory function improved via the use of sensory aids. Furthermore, one study included in the review has highlighted the importance of considering DSI onset as dementia risk increased for every additional year with DSI [46]. However, all studies included in the review, assessed sensory function only at baseline with no follow-up measurements included. Furthermore, none of the studies considered participants’ use of sensory aids, which may impact the association between sensory function and dementia. Future research should therefore incorporate follow-up assessments of sensory function, and account for improvements and declines, as well as the timing of DSI onset.

Although most studies assessed sensory impairments using subjective measures, the literature revealed a lack of validated measurement tools as well as significant variability in reported outcomes. Notably, participants across the included studies were more likely to report sensory impairment(s) when using subjective rather than objective measurements. This finding could be attributed to several factors. Firstly, when considering nonspecific sensory function questions (i.e. ‘Do you have trouble seeing/hearing?’) may lead to variability in participants’ points of reference, with some participants comparing their abilities to peers and others to past sensory capacities [62]. Additionally, previous research has reported that self-reported HI or VI increases the likelihood of reporting impairment in the other sense [63]. This may be because vision and hearing complement each other in activities like socialisation, which are often used to self-assess sensory function [63]. Similarly, cognitive impairment itself can impact activities used for sensory self-evaluation, such as being able to follow conversations [64–67]. These difficulties resulting from cognitive decline may be misattributed to sensory difficulties [64–66]. This theory is supported by our data indicating that participants with dementia reported more sensory dysfunction when subjective rather than objective measurements were used, as well as by the increased heterogeneity of the meta-analysis sub-analysis focusing exclusively on subjective measurements. However, since only five studies using objective measurements reported sufficient prevalence data for comparison with those using subjective assessments, and none employed a combination of objective and subjective measurements, these findings should be interpreted with caution. Furthermore, our findings of higher subjective sensory impairment in those with later dementia could be explained by the fact that participants could have been in early stages of dementia at the point of sensory measurement and that early cognitive changes might have impacted their self-evaluation. However, this is somewhat challenged by an unchanged HR of the meta-analysis when studies with a follow-up of <5 years were excluded. Finally, it should be noted that other factors like age [67–69], gender [70], and ethnicity [71] have been found to affect accuracy of self-rated sensory abilities, and may have affected prevalence of SSI and DSI in the included studies.

Nevertheless, if cognitive decline affects subjective sensory function, it raises the question whether subjective assessments truly reflect sensory status or instead serve as indicators of cognitive function. However, other researchers have argued that acuity measurements may oversimplify sensory function, with subjective measurements offering a more accurate reflection of everyday sensory function [62, 70, 72]. This concern, however, could be mitigated by the use of behavioural tests that combine sensory abilities [73, 74]. Indeed, one study reported that dementia risk was greater when considering global sensory function, rather than isolated acuity measures [75]. In this regard, measures of multisensory integration—which assess how individuals combine information from multiple sensory modalities—could provide valuable insights into the relationship between sensory function and cognition. Such measures offer a more comprehensive understanding of how the combined performance of sensory systems influences cognitive process and daily functioning. Notably, older adults exhibit less precision in combining audiovisual information compared to younger adults [76–79], with this impairment being more pronounced in those experiencing cognitive decline [80, 81]. Thus, multisensory integration appears to be a strong candidate for providing an objective measurement of sensory performance that is sensitive to changes in cognitive function.

In conclusion, this review demonstrates a robust association between DSI and dementia, with evidence suggesting that DSI may pose a greater risk factor for dementia than SSI. Although causality between DSI and dementia cannot be established, the elevated dementia hazard linked to DSI suggests that DSI might be an early, modifiable risk factor for dementia and could potentially be used for early dementia detection. However, the present review also highlighted several limitations within the DSI and dementia literature, including absence of measurement validation, consideration of multisensory interactions, use of sensory aids, sensory impairment onset and potential impacts of sensory difficulties on cognitive tests. Future research should address these limitations and further examine whether sensory aids and other compensatory strategies might help mitigate cognitive decline in individuals with DSI.

## Supplementary Material

aa-25-0767-File002_afaf267
