# Supplementary material for: The association between dual sensory impairment and dementia: a meta-analysis and systematic review of the literature
Source: Age Ageing. 2025 Sep 27;54(9):afaf267. doi: 10.1093/ageing/afaf267 (PMC12476137; doi:10.1093/ageing/afaf267)
Supplement: aa-25-0767-File002_afaf267 [file aa-25-0767-file002_afaf267.docx]

**Supplementary Material:***The association between dual sensory impairment and dementia: A meta-analysis and systematic review of the literature*

Table of Contents

[Appendix 1 - Detailed Methodology 2](#_Toc206926127)

[Appendix 2 – Prisma Flow Chart 7](#_Toc206926128)

[Appendix 3 – Subjective and Objective Sensory Measurements 8](#_Toc206926129)

[Appendix 4 - Dementia and sensory impairment prevalence 14](#_Toc206926130)

[Appendix 5 - Dementia Types 16](#_Toc206926131)

# Appendix 1 - Detailed Methodology

*Inclusion criteria*

We used the PICOS tool to determine the inclusion and exclusion criteria for the systematic review (see Table S1). The population (P) of interest was individuals with DSI, consisting of concurrent HI and VI, who were over the age of 18. Subjective and objective measurements of sensory impairment were included. Corrective interventions (I) for sensory impairment (e.g., glasses, hearing aids) were included. Studies implementing interventions to target cognitive decline were excluded. The comparison group (C) consisted of individuals with NSI or those with SSI. The outcome (O) of interest was objectively measured dementia incidence or prevalence. Studies that measured severe cognitive impairment but did not refer to dementia diagnosis were not included. This decision was made to ensure the review remains focused on confirmed cases of dementia. Considering study design (S), we included peer-reviewed articles published in English. Reviews, letters, dissertations, book chapters, opinion pieces, conference abstracts, protocols, case studies, animal studies and editorials were excluded.

**Table S1**. Inclusion and Exclusion Criteria according to PICOS

|  | Inclusion Criteria | Exclusion Criteria |
| --- | --- | --- |
| **P**opulation of Interest | - Adults over the age of 18 - Participants with audiovisual DSI - Subjective or objective measurement of sensory impairment | - Children and adolescents - Participants with DSI in sensory modalities other than auditory and visual |
| **I**ntervention | - Glasses and hearing aids - Medication and supplements for dementia | - Any intervention directly targeting cognitive decline |
| **C**omparisons | - Participants with either no sensory impairment, or single sensory impairment (HI / VI only) | ● No comparison between the control groups and the DSI groups in terms of dementia/cognition is made |
| **O**utcome | - Dementia - Objective measurement of cognition (e.g., MMSE, MOCA, DSM criteria) - Self-reported physician diagnosis (e.g., ‘Has a doctor ever told you that you have dementia?’) | - Subjective measurements of cognition (e.g., self-reported cognition) |
| **S**tudies | - Peer-reviewed studies - Available in or translated to English - Longitudinal or cross-sectional | - Reviews, grey literature, meta-analyses, case reports, animal studies, commentaries, editorials, book chapters, opinion pieces |

*Search Strategy*

Database searches were conducted on the 29th of February and on the 30th of August 2024 on PsycINFO PubMed, and Web of Science. The search strategy included the following terms:

Search 1: Visual Impairment

“Vision disorder*” OR “visual disorder*” OR “visual acuity” OR “vision acuity” OR “vision loss” OR “visual loss” OR “seeing loss” OR “visual impairment*” OR “vision impairment*” OR blindness OR blindsight OR “eye disorder*” OR hemianopia OR “low vision”

Search 2: Auditory Impairment

“Hearing impairment*” OR “auditory impairment*” OR “hearing loss” OR deaf* OR presbycusis OR “hearing disorder*”

Search 3: Audiovisual Impairment

S1 AND S2

Search 4: Dual Sensory Impairment

“Dual sensory impairment” OR “multisensory impairment” OR “audiovisual impairment” OR “multi-sensory impairment*” OR “multi sensory impairment*” OR “multisensory integration” OR “multi-sensory integration”

Search 5: Audiovisual Dual Sensory Impairment

S3 OR S4

Search 6: Dementia & CI

Dementia OR Alzheimer* OR “Vascular Dementia” OR “Dementia with Lewy Bodies” OR “Lewy Body” OR “Mild Cognitive Impairment” OR MCI OR “Cognitive Dysfunction” OR “Cognitive Decline” OR "Frontotemporal Lobar Degeneration" OR "Senile Dementia" OR “Early Onset Dementia” OR “Frontotemporal Dementia”

Search 7: Final Search

S6 AND S5

*Screening of Studies*

All references were uploaded to the online systematic review management tool Covidence for duplicate removal and screening. Both reviewers (NMZ and KB) independently screened abstracts and full texts of the selected studies.

*Quality Appraisal*

In order to evaluate the risk of bias of included articles, a 12-item quality appraisal tool was applied. NMZ applied a score of ‘2’ to items rated with ‘yes’, a score of ‘1’ for items marked ‘partially’, as well as a score of ‘0’ for items rated ‘no’. Articles that scored a total of 17 to 24 points across items were considered good quality,

*Data Extraction*

Data extraction was completed by the first author in a Google Sheets spreadsheet. Extracted information included: Article title; author names; year of publication; study location; study aims; study design; parent project or identifier (if applicable); number of participants overall and per sensory impairment group (baseline and follow-up, if applicable); participant characteristics; mean age and age range; gender/sex distribution; covariates; HI and VI measurement and cut-off; DSI definition; inclusion/exclusion of corrective aids (and grouping if applicable); use and grouping of corrective aids users during testing; availability of sensory impairment onset measurement; dementia measurement and cut-off; number of participants with dementia overall and per sensory impairment group (baseline and follow-up, if applicable); statistical analysis; key results and implications. For studies reporting the hazard ratio (HR) of dementia in sensory impairment groups, both the HR and corresponding 95% confidence intervals (CI) were recorded. If measurement of sensory impairment was not described in sufficient detail, or it was not stated if participants wore sensory aids during testing, NMZ contacted the authors of the study for further information. However, studies remained included in the review, even if authors did not respond to the request.

*Statistical Analysis and Data synthesis*

To conduct a statistical analysis of results, HRs and 95% CIs were extracted. In studies reporting multiple models with covariates included, we used the HR derived from the model with the most covariates included. Standard errors were estimated from the 95% CIs, and the log transformation of HRs was conducted for normalisation of the distribution. To test for heterogeneity, Cochrane’s Q test, as well as the I^2^ statistic were calculated, with results below 50% indicating moderate heterogeneity [29]. Furthermore, to compare the dementia risk between DSI, HI, and VI groups, the relative excess risk due to interaction was calculated using the following equation [30]:

RERI= HRDSI - HRHI - HRVI +1

Moreover, sensitivity analyses were conducted to examine the influence of each study on the overall findings by sequentially excluding individual studies from the model. Publication bias was assessed by examining funnel plots. Forest plots were created to illustrate the individual and pooled effect size of all studies included in the meta-analysis.

Studies that did not report hazard ratios were excluded from the meta-analysis. Instead, these studies were summarised through narrative synthesis, Graphs of relevant results were created using Graph Prism.

# Appendix 2 – Prisma Flow Chart

Figure S1. Prisma Flow Chart of Included and Excluded Studies**.**


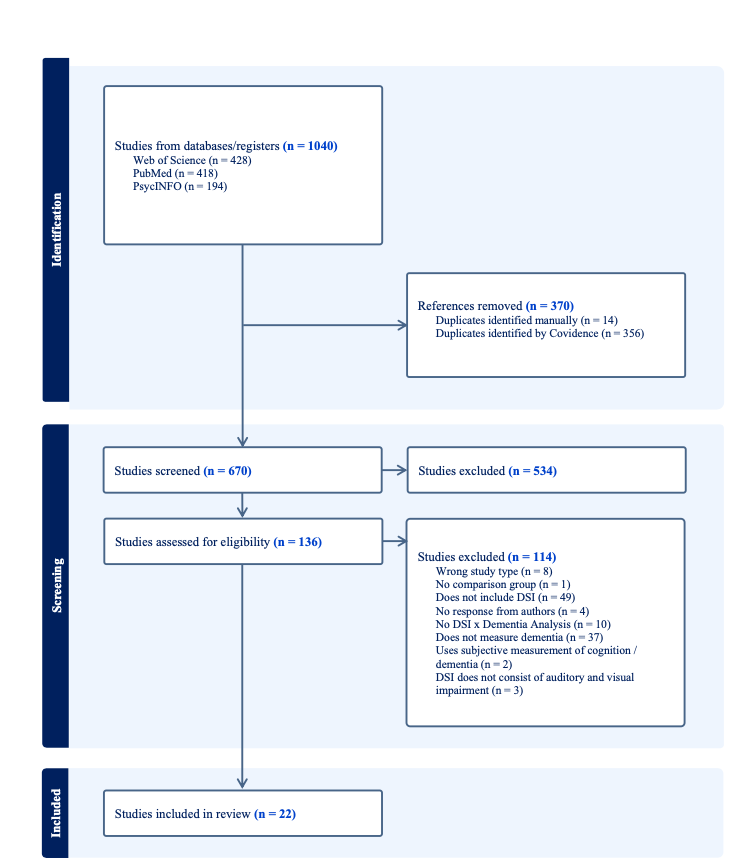


# Appendix 3 – Subjective and Objective Sensory Measurements

***Assessment of Sensory Impairments***

*Visual Impairment*

Of the 22 included studies, VI was most commonly measured using subjective assessment (n=14) [21,23,24,31–33,35,37,38,40,42,45–47]. More specifically, subjective assessment of VI assessment included self-report (n=11), a combination of self-report and assessor report, including reports from healthcare care professionals or researchers and physicians (n=3) as well as assessor report only (n=1) (see Table S2).

Of the 8 studies assessing vision objectively [25,26,34,36,39,41,43,44], most assessed visual acuity (n=5) using the Snellen chart (n=2) or the LogMar chart (n=2). One study did not specify which visual acuity chart was used to assess visual acuity. Two studies assessed VIvisual acuity using ICD-10 codes for visual disorders. Furthermore, one study assessed visual field, and another study assessed near vision using the Jaeger Eye chart (see Table S3).

**Table S2** – Overview of Subjective Vision Measurements

| Outcome | Situation/Question | Assessment Type | Scale | Cut off | Corrective Aid Use | Used by (Citation) |
| --- | --- | --- | --- | --- | --- | --- |
| General Vision | General vision / ‘How good is your eyesight?’ | Self-report | ‘excellent’, ‘very good’, ‘good’, ‘fair’, ‘poor’ | ‘fair’ or ‘poor’ | Aided | Li et al., 2024, Maharani et al., 2020 |
|  | Vision problems / “Difficulty seeing” | Self-report | No impairment, slight impairment, moderate impairment, severe/profound impairment | Slight, moderate, or severe/profound impairment | Not reported | Pabst et al., 2021, |
|  |  |  | ‘little trouble’ or ‘a lot of trouble’ | ‘lot of trouble’ | Not reported | Deardorff et al., 2019 |
|  | Identifying objects | Self-report | ‘yes’, ‘no’ | ‘no’ | Aided | Davidson & Guthrie 2019, Yamada et al. 2014, Yamada et al., 2016 |
|  | Driving | Self-report | ‘yes’, ‘no’ | ‘no’ | Aided | Hwang et al., 2020, Hwang et al., 2022 |
| Near Vision | Reading newspaper (print) | Self-report | ‘yes’, ‘no’ | ‘no’ | Aided | Assi et al. 2021, Davidson & Guthrie 2019, Hwang et al., 2020, Hwang et al., 2022, Kuo et al., 2021, |
|  |  |  | ‘reduced with glasses/lenses’, ‘reduced without glasses/lenses’ | ‘yes’ | Aided | Byeon et al., 2021 |
|  |  |  | ‘excellent’, ‘very good’, ‘good’, ‘fair’, ‘poor’ | ‘fair’ or ‘poor’ | Aided | Möller et al., 2024 |
|  | Reading newspaper (headlines) | Self-report | ‘yes’, ‘no’ | ‘no’ | Aided | Davidson & Guthrie 2019, Yamada et al. 2014, Yamada et al., 2016 |
| Distance Vision | Recognise someone across the street/room | Self-report | ‘yes’, ‘no’ | ‘no’ | Aided | Assi et al. 2021, Hwang et al., 2020, Hwang et al., 2022, Kuo et al., 2021, |
|  |  |  | ‘excellent’, ‘very good’, ‘good’, ‘fair’, ‘poor’ | ‘fair’ or ‘poor’ | Aided | Möller et al., 2024 |
|  | Watching television | Self-report | ‘yes’, ‘no’ | ‘no’ | Aided | Hwang et al., 2020, Hwang et al., 2022 |
| Blindness | N/a | Self-report | ‘yes’, ‘no’ | ‘yes’ | n/a | Assi et al. 2021, Kuo et al., 2021, |
|  |  | Assessor report | ‘severe impairment, including blindness. Blind or can only distinguish lights, colours, or shapes, but does not follow objects’ | ‘yes’ | Aided | Yamada et al. 2014, Yamada et al., 2016 |

**Table S3** – Overview of Objective Vision Measurements

| Outcome | Assessment Type | Specific Assessment | Cut Off | Measured eye | Corrective Aid Use | Used By |
| --- | --- | --- | --- | --- | --- | --- |
| Visual acuity (Distance Vision) | Chart | Snellen chart | 20/40 / > 0.3 logMAR | Both | Aided | Oh et al., 2023 |
|  |  |  | Control group median | Better | Aided | Uhlmann et al., 1991 |
|  |  | LogMar chart | > 0.3 logMAR | Better | Aided | Hu et al., 2022, Shi et al., 2024 |
|  | Not reported | Not reported | <0.05 | Better | Aided | Luo et al., 2018 |
| Near Vision | Chart | Jaeger Eye chart | ≥ J2 | Both (Average) | Aided | Dintica et al. 2023 |
| Visual Field | Not reported | Not reported | Visual field less than 10 degrees | Better | Not reported | Luo et al., 2018 |
| Medical diagnosis | Medical files | ICD |  | n/a | n/a | Kim et al., 2024, Michalowksy et al., 2019, |

*Hearing Impairment*

Overall, 15 of the included studies measured HI subjectively [21,23,24,31–33,35,37,38,40,42,43,45–47]. The majority relied on self-report assessments (n=14). Only one study made use of assessor reports. The most common scenarios to measure HI subjectively included ability to hear conversation in a crowded room (n=4) or when the TV or radio is playing (n=2). Five studies asked participants if they were able to use the telephone. Two studies assessed whether a speaker had to adjust their voice to be understood. A total of three studies asked about hearing aid use as an indicator of HI, and nine studies considered deafness (see Table S4).

Of the 7 studies measuring hearing ability objectively [25,26,34,36,39,41,44], a majority measured relied on the measurement of hearing thresholds to assess HI, with three studies employing pure-tone audiometry. Other methods of objective assessment included checking medical files for hearing-related ICD codes relating to hearing disorders (n=2). One study checked for ICD codes indicating HI, as well as using self-report and one study used the digit triplet test (see Table S5).

**Table S4** – Overview of Subjective Hearing Measurements

| Outcome | Assessment Type | Specific Assessment | Cut Off | Measured eye | Corrective Aid Use | Used By |
| --- | --- | --- | --- | --- | --- | --- |
| Visual acuity (Distance Vision) | Chart | Snellen chart | 20/40 / > 0.3 logMAR | Both | Aided | Oh et al., 2023 |
|  |  |  | Control group median | Better | Aided | Uhlmann et al., 1991 |
|  |  | LogMar chart | > 0.3 logMAR | Better | Aided | Hu et al., 2022, Shi et al., 2024 |
|  | Not reported | Not reported | <0.05 | Better | Aided | Luo et al., 2018 |
| Near Vision | Chart | Jaeger Eye chart | ≥ J2 | Both (Average) | Aided | Dintica et al. 2023 |
| Visual Field | Not reported | Not reported | Visual field less than 10 degrees | Better | Not reported | Luo et al., 2018 |
| Medical diagnosis | Medical files | ICD |  | n/a | n/a | Kim et al., 2024, Michalowksy et al., 2019, |

**Table S5** – Overview of Objective Hearing Measurements

| Outcome | Assessment Type | Specific Assessment | Cut Off | Measured eye | Corrective Aid Use | Used By |
| --- | --- | --- | --- | --- | --- | --- |
| Visual acuity (Distance Vision) | Chart | Snellen chart | 20/40 / > 0.3 logMAR | Both | Aided | Oh et al., 2023 |
|  |  |  | Control group median | Better | Aided | Uhlmann et al., 1991 |
|  |  | LogMar chart | > 0.3 logMAR | Better | Aided | Hu et al., 2022, Shi et al., 2024 |
|  | Not reported | Not reported | <0.05 | Better | Aided | Luo et al., 2018 |
| Near Vision | Chart | Jaeger Eye chart | ≥ J2 | Both (Average) | Aided | Dintica et al. 2023 |
| Visual Field | Not reported | Not reported | Visual field less than 10 degrees | Better | Not reported | Luo et al., 2018 |
| Medical diagnosis | Medical files | ICD |  | n/a | n/a | Kim et al., 2024, Michalowksy et al., 2019, |

*Dual Sensory Impairment*

Most studies defined DSI as the presence of HI and VI according to the respective criteria or cut-off (n=21). Only one study made use of the deafblind severity index score ≥ 3 as an indicator for DSI [33].

# Appendix 4 - Dementia and sensory impairment prevalence

Dementia prevalence ranged from 0.5% [[36]](https://www.zotero.org/google-docs/?bT8l43) to 52.6% [[38]](https://www.zotero.org/google-docs/?IGLdvn). Specifically, of the 1,165,580 participants with prevalence data reported included, 184,202 were reported to have dementia (weighted average=15.80%, weighted SD=16.61).

Of those with DSI, 28579 were identified as having dementia (weighted average=29.23%, weighted SD=12.09). Dementia prevalence of participants with SSI was slightly lower (weighted average=17.02%, weighted SD=20.88), while dementia prevalence was lowest in those individuals with NSI (weighted average=11.14%, weighted SD=7.19) (see Figure S2).

**Figure S2**. Prevalence of dementia in the no sensory impairment (NSI), single sensory impairment (SSI) and dual sensory impairment (DSI) groups of all studies reporting both dementia and sensory impairment prevalence at baseline, as well as weighted average percentage across all studies. Error bars above and below the mean indicate standard error of the mean.


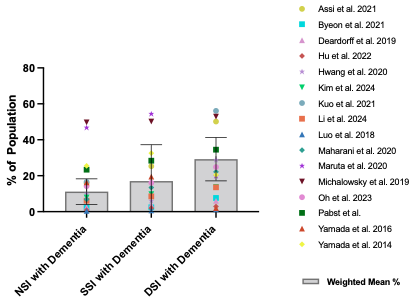


Of those participants with dementia, DSI prevalence ranged from 1.38% [[41]](https://www.zotero.org/google-docs/?UJ3i1D) to 62.72% [[31]](https://www.zotero.org/google-docs/?jupVFv). Specifically, out of 1,156,939 participants with both dementia and DSI prevalence reported, 25,471 were identified as having DSI (weighted average= 15.74%, weighted SD=9.38). Moreover, prevalence of SSI in the dementia group ranged from 23.95% [[34]](https://www.zotero.org/google-docs/?OEiUM4) to 37.14% [[42]](https://www.zotero.org/google-docs/?l5d06K) (weighted average= 29.24%, weighted SD=5.28). Lastly, between 7.1% [[31]](https://www.zotero.org/google-docs/?7LRluf) and 72.86% [[34]](https://www.zotero.org/google-docs/?IUPuiT) of participants with dementia did not have a sensory impairment (weighted average= 64.42%, weighted SD=7.85).

More participants with dementia reported SSI (weighted average= 72.33%, weighted SD=6.21) as well as DSI (weighted average=22.91%, weighted SD=1-20) when they were asked to self-report sensory function compared to when participants underwent behavioural assessment such as pure tone audiometry or visual acuity charts (SSI: weighted average= 28.29%, weighted SD=0.27 , DSI: weighted average= 4.94%, weighted SD=0.45).

# Appendix 5 - Dementia Types

A total of eleven studies considered Alzheimer’s dementia (AD). Of those studies, five found an association between DSI and AD [[21,34,44,46,47]](https://www.zotero.org/google-docs/?x9zDVQ) while one study reported greater prevalence of AD in the DSI group [[45]](https://www.zotero.org/google-docs/?ZSI4Bf). Only one of these studies found an association between SSI and AD as well [[46]](https://www.zotero.org/google-docs/?AAZSnG). Moreover, one study found an association between VI and AD and VI, but no association between DSI or HI and AD risk [[43]](https://www.zotero.org/google-docs/?HxIVDo). Two studies only considered AD but not all-cause dementia and reported an increased risk in the DSI group compared to both NSI and SSI [[34,44]](https://www.zotero.org/google-docs/?TG6ZWS). Two studies found no association between DSI and AD, but an association with all-cause dementia [[33,43]](https://www.zotero.org/google-docs/?WNDPc1) and two found no association for either dementia type [[23,31]](https://www.zotero.org/google-docs/?XfHQIb). Three studies considered vascular dementia, of which two found no increased risk in the DSI or SSI group [[21,46]](https://www.zotero.org/google-docs/?yJbwY3). Only one study found a positive association between DSI and vascular dementia risk [[43]](https://www.zotero.org/google-docs/?AV1adW). The study also found an increased risk for vascular dementia in the HI and VI group, and the risk for vascular dementia was greatest for those participants with VI only.
